# Varicella zoster virus Vasculopathy as a Rare Cause of Stroke

**DOI:** 10.1590/0037-8682-0215-2023

**Published:** 2023-07-24

**Authors:** Fatma Şimşek, Alper Eren, Nazım Kızıldağ

**Affiliations:** 1 Ataturk University, Faculty of Medicine, Department of Neurology, Erzurum, Turkey. Ataturk University Faculty of Medicine Department of Neurology Erzurum Turkey

**FIGURE 1:**
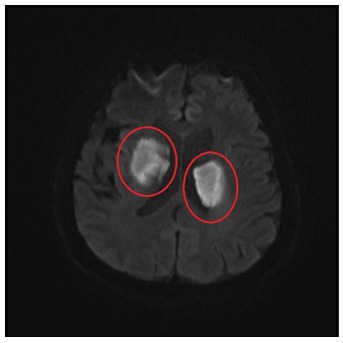
Hyperintense areas in bilateral suprathalamic regions in B1000 sequence on diffusion-weighted MRI.

**FIGURE 2:**
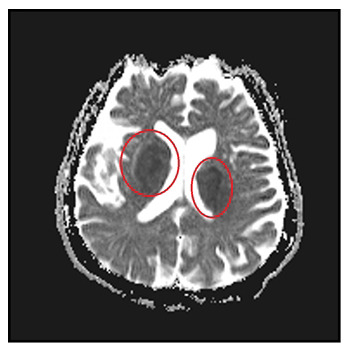
Hypointense areas consistent with diffusion-limiting ischemia in bilateral suprathalamic regions on diffusion-weighted MRI ADC sequence.

A 72-year-old male patient presented with a decreased level of consciousness. He had no known history of systemic disease. He also had a painful vesicular rash on the scalp three weeks prior that had resolved. His neurological examination, during which he could not respond to verbal stimuli, revealed that his four extremities responded to painful stimuli and that the plantar reflex was downgoing bilaterally. His body temperature was 37.3°C. Diffusion-weighted magnetic resonance imaging (MRI) revealed infarcted areas, indicating diffusion restriction in both suprathalamic regions (Figures 1 and 2). A lumbar puncture was performed. No cells were observed upon direct examination of the cerebrospinal fluid. The microprotein levels were high at 65.4 mg/dL. Polymerase chain reaction (PCR) for varicella zoster virus (VZV) DNA was positive. Serum VZV IgG >1500 mIU/mL was positive, and serum VZV IgM was 2.2 mIU/mL was negative. The patient was given 10 mg/kg acyclovir three times daily for 21 days and 60 mg/kg methylprednisolone for 14 days. The patient began to respond significantly to verbal stimuli in the fourth month of hospitalization. VZV can spread centrally from the peripheral nerves, causing neurovascular disorders ranging from ischemic stroke-related large-vessel vasculopathy to arterial dissection and subarachnoid hemorrhage^1^. VZV vasculopathy is often chronic and long-lasting. Clinical improvement is possible with antiviral therapy^2^. Given that VZV is a treatable cause of stroke, its etiology should be investigated even if the rash is not described.
